# Isotopic ecology of coyotes from scat and road kill carcasses: A complementary approach to feeding experiments

**DOI:** 10.1371/journal.pone.0174897

**Published:** 2017-04-03

**Authors:** Rachel E. B. Reid, Paul L. Koch

**Affiliations:** Earth and Planetary Sciences Department, University of California, Santa Cruz, California, United States of America; Stockholm University, SWEDEN

## Abstract

Scat is frequently used to study animal diets because it is easy to find and collect, but one concern is that gross fecal analysis (GFA) techniques exaggerate the importance of small-bodied prey to mammalian mesopredator diets. To capitalize on the benefits of scat, we suggest the analysis of scat carbon and nitrogen isotope values (δ^13^C and δ^15^N). This technique offers researchers a non-invasive method to gather short-term dietary information. We conducted three interrelated studies to validate the use of isotopic values from coyote scat: 1) we determined tissue-to-tissue apparent C and N isotope enrichment factors (ε^13^* and ε^15^*) for coyotes from road kill animals (n = 4); 2) we derived diet-to-scat isotope discrimination factors for coyotes; and 3) we used field collected coyote scats (n = 12) to compare estimates of coyote dietary proportions from stable isotope mixing models with estimates from two GFA techniques. Scat consistently had the lowest δ^13^C and δ^15^N values among the tissues sampled. We derived a diet-to-scat Δ^13^C value of -1.5‰ ± 1.6‰ and Δ^15^N value of 2.3‰ ± 1.3‰ for coyotes. Coyote scat δ^13^C and δ^15^N values adjusted for discrimination consistently plot within the isotopic mixing space created by known dietary items. In comparison with GFA results, we found that mixing model estimates of coyote dietary proportions de-emphasize the importance of small-bodied prey. Coyote scat δ^13^C and δ^15^N values therefore offer a relatively quick and non-invasive way to gain accurate dietary information.

## Introduction

Scat is ubiquitous and easy to sample and has therefore historically formed our perception of mammalian carnivore and omnivore dietary ecology [1]. The dissection, identification, and quantification of the material contained in scats is labor intensive and time consuming and can be hampered by observer bias [2] as well as discrepancies among diet quantification methods [1]. Gross fecal analysis (GFA) techniques are also likely to severely overestimate the importance of small diet items [3–6]. Stable isotope analyses of scat have the potential to provide a quick and possibly more accurate means of gaining dietary information from scat while also allowing for non-invasive isotopic investigation of short-term dietary shifts that might otherwise be masked by long-term averaging in other animal tissues [7–14].

Because scat is composed of a combination of undigested food, sloughed epithelial cells, and microbiota [15,16], there is concern that the poorly digested food in scat will disproportionally affect its isotopic values. Scat has therefore largely been excluded from controlled feeding studies, which are often used to determine species- and tissue-specific discrimination factors (the difference in isotopic composition between a tissue, such as hair, and an animal’s diet), which are essential for the accurate determination of dietary sources. Scat is, however, an ideal substrate; it is abundant, turns over rapidly, and can be collected opportunistically without needing to capture the target animal. Both feeding studies [9,17–20] and field tests [7,12,13,21,22] support the notion that scat stable isotope values do reflect ingested diet for many organisms. Most of these studies have focused on mammalian herbivores rather than omnivores or carnivores. To our knowledge, the only non-herbivorous mammals to have their scats characterized isotopically are bears [12], mountain gorillas [7], chimpanzees [23], bats [24], and two species of big cats [25].

**Fig 1:**
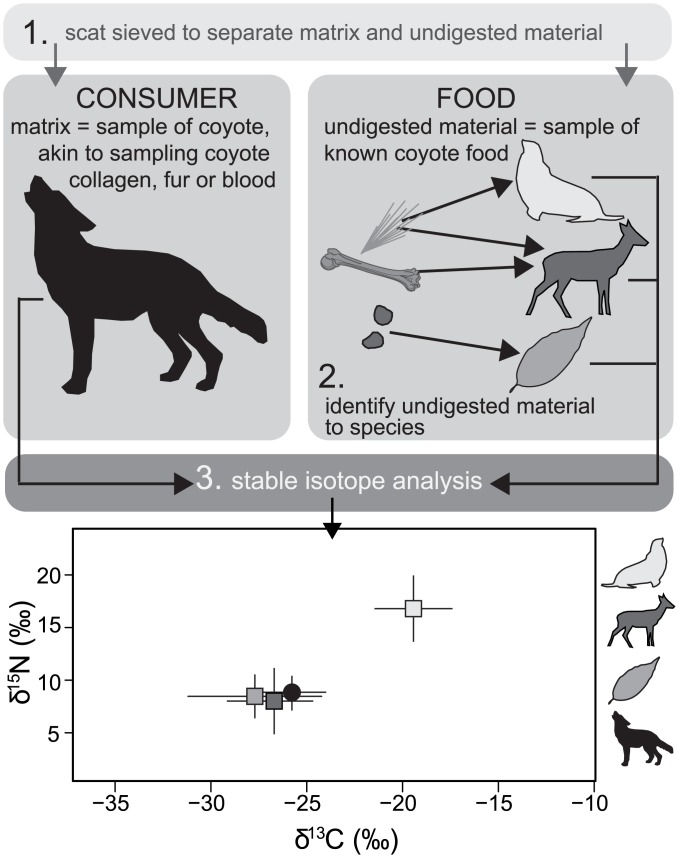
Scat stable isotope sampling rationale. Step 1 is to separate the coyote scat—the fine-grained material binding the scat together—from the clearly undigested scat components. In step 2, the undigested materials are identified to the finest taxonomical level possible. In step 3, we conduct stable isotope analyses of both the coyote (scat matrix) and its known diet (identified undigested material). After correcting scat values for diet-to-scat discrimination, we expect that they should fall within the mixing space created by known dietary items.

Here, we determine isotopic differences among coyote tissues (hereafter termed apparent enrichment, ε*, defined below) and ground truth scat carbon and nitrogen isotope values (δ^13^C and δ^15^N) as dietary proxies for coyotes (*Canis latrans*), one of the most abundant and ecologically-impactful carnivores in North and Central America. As long as the difference in timescale over which disparate tissues integrate diet is considered, tissue-to-tissue apparent enrichment factors are valuable tools that allow for comparison among studies that rely on different tissues. For example, bone collagen is often the tissue of choice for studies using historical and archaeological materials because it preserves relatively well over long timescales. Modern studies, on the other hand, often focus on more easily and ethically sampled tissues, such as hair, whiskers, or blood. We took a novel approach, choosing to characterize isotopic differences among coyote tissues by analyzing materials from individual road kill carcasses. One advantage to this approach is that it enables sampling of tissues that are impossible to non-invasively collect from living animals (e.g., bone collagen). It is also less resource intensive than a feeding study on animals in a controlled setting. The downsides to our approach are that we are limited to the small number of individual animals that were collectible during our study period and, more challengingly, we do not have direct measurements of the diets of road kill animals. Therefore, in order to derive diet-to-scat δ^13^C and δ^15^N discrimination factors for coyotes, we rely on previous results from controlled feeding studies on two other wild canid species: red foxes [26] and wolves [27]. We tested our derived diet-to-scat discrimination factors by applying them to fully dissected coyote scats (DNA-verified to species) and compared them with isotope values measured in known dietary items. We hypothesize that the coyote scat matrix—the material that binds the scat together—primarily contains material derived from the coyote itself (e.g., epithelial cells) and, once corrected for discrimination, scat stable isotope values will fall within the isotopic mixing space created by known dietary items (Fig 1). Finally, we compare quantitative estimates of coyote dietary proportions derived from Bayesian stable isotope mixing models with estimates of diet composition from GFA to determine whether an isotopic approach mitigates some of the known biases in GFA. We present C and N isotope data from multiple tissues sampled from four road kill coyote carcasses and from seasonally collected coyote scats that are DNA-verified to species.

## Materials and methods

### Sample collection

We used road kill carcasses for the determination of coyote tissue-to-tissue enrichment factors. We collected road kill coyotes under California Fish and Game permit SC-11995 to R. Reid. All work with animals followed the guidelines of the American Society of Mammalogists [28] and was conducted with the approval of both the UC Santa Cruz Institutional Animal Care and Use Committee (permits Kochp1105 and Kochp1211) and the Office of Environmental Health and Safety. Specimens were collected fresh and stored frozen until dissection, thereby preventing tissue deterioration.

We collected fresh coyote scats quarterly along a ~6 km coast-to-inland transect in 2011–2013 at Año Nuevo State Park and Reserve (San Mateo County, CA). As with the carcass samples, our scat collection followed all applicable institutional and national guidelines for the care and use of animals. Our field collection methods are described in full in [29]. Briefly, we cleared all scats from the transect one week prior to collecting all scats deposited in the intervening week. We recorded the scat locations with a GPS and stored the scats in the freezer in individually labeled Ziploc^®^ bags. Scats deposited on the ground will experience weathering and decomposition at rates determined by both the local environment and the composition of the scat [30]. Previous research on wolf scats shows that scat DNA degrades more rapidly in samples that are in direct contact with soil, likely because they are kept wet, which may facilitate their invasion by decomposers (e.g., bacteria, fungi, insects) [31]. While plant δ^13^C and δ^15^N values have been shown to change over weeks to months during decomposition (e.g., [32,33]), given that we collected all scats within a week of their deposition, we argue that any potential changes in isotopic values associated with decomposition are not relevant to these samples.

### Isotopic analyses

We sampled fur, muscle, bone, and feces from each animal carcass. We clipped a small tuft of fur from each animal's dorsum. Muscle tissue was consistently sampled from the right trapezius. We defleshed and removed a small chip of bone (~50 mg) for collagen extraction; if the carcass did not have easily sampled broken bones, we defaulted to a chip from the mandible. We sampled feces from what remained in the colon. Hair samples were rinsed with Milli-Q water, immersed in petroleum ether and sonicated for 15 minutes, rinsed again with Milli-Q water and dried in a 60°C oven overnight [34]. Our collagen extraction methods followed Brown et al. [35]. We decalcified bone fragments in 0.5N HCl for ~72 hours then followed with a 0.1N NaOH treatment for 24 hours to saponify fatty acids. We rinsed the samples five times in Milli-Q water, lipid extracted them in the same manner as the muscle tissue, rinsed them 5x with Milli-Q, and then freeze dried them overnight. Muscle samples were freeze-dried overnight and ground to a powder in an agate mortar and pestle. We lipid extracted muscle tissue and bone collagen by immersing the samples in 5 mL of petroleum ether [36] and sonicating for 15 min 2–3 times. Then, we rinsed 5 times in Milli-Q water before freeze drying the samples overnight. Muscle was re-homogenized and, like the collagen and hair samples, we weighed ~0.7 mg of material into 5 x 9-mm tin capsules. The atomic C/N ratio in the bone collagen samples fell between 2.8 and 3.4, which is within the range expected for modern collagen [37].

To prepare scat samples for isotopic analysis, we isolated the scat matrix—the material that binds the scat together—because we anticipated that this portion of the scat primarily contains material derived from the coyote itself (e.g., epithelial cells). By excluding poorly digested dietary components, we also prevent those items from having a disproportional effect on the scat stable isotope values. We extracted the scat matrix material by gently breaking apart oven-dried scats over a fine mesh sieve (0.420 mm). The matrix passes through the sieve while other scat components, such as fur, feathers, or bone are captured in the sieve. We then cleaned the matrix by placing it in filter paper cones and rinsing it first with Milli-Q water, then with 0.1N HCl to remove possible carbonate contaminants (e.g. bone fragments), and then again with Milli-Q. Because previous authors conjectured that rinsing with distilled or Milli-Q water following acidification may introduce bias in sample δ^15^N values [38,39], we characterized this possible bias by comparing 7 paired samples that were (a) rinsed following acidification or (b) not rinsed following acidification. We found no significant difference between the δ^15^N values of the two sample treatment groups (paired t-test: mean sample difference = -0.11‰, df = 6, *p* = 0.055; S1 Fig). After the scat samples were fully dry and homogenized, we weighed approximately 5 mg of scat matrix into 5 x 9-mm tin boats for isotopic analysis. Scats were identified to species with mitochondrial DNA [29].

To enable comparison between scat matrix and isotopic values of known diet components, we cleaned and analyzed a subset of identified scat components. We prepped hair and bone samples for isotopic analyses as described above. We prepped feather samples in the same way as hair. Arthropod, vegetation, and seed samples were repeatedly rinsed and sonicated in Milli-Q water (4x for 15 min), dried (60°C overnight), and then crushed with an agate mortar and pestle. We weighed ~0.7 mg of hair, feather, collagen, and arthropod samples into 5 x 9-mm tin boats. We divided vegetation samples into aliquots of ~0.4 mg for carbon isotopes and ~3 mg for nitrogen isotopes, and sealed them in 5 x 9-mm tin boats.

All samples were combusted via Dumas combustion using a Carlo Erba 1108 elemental analyzer and analyzed for δ^13^C and δ^15^N values on a ThermoFinnigan Delta Plus XP continuous-flow, isotope-ratio-monitoring mass spectrometer at the UC Santa Cruz Stable Isotope Laboratory. We report our results using δ notation, in which δ^H^X = ((R_sample_/R_standard_)– 1) x 1,000, where R is the ratio of the heavy isotope to the light isotope for element X. Carbon isotope values are reported relative to Vienna Pee Dee Belemnite (a marine carbonate) and nitrogen isotope values are reported relative to air, and the resulting value is expressed in parts per thousand (i.e., per mil, ‰). Sample isotopic values are corrected for size, drift and source stretching effects. The average analytical precision was <0.2‰ for both carbon and nitrogen, based on the *SD* of 41 replicates of an in-house standard (PUGel) and 20 replicates of a second in-house standard (Acetanilide). Atomic carbon and nitrogen elemental composition is estimated based on standards of known elemental composition (PUGel and Acetanilide) and precision of these known compounds is determined to better than 1%.

Often the offset, or fractionation, between two substances or tissues is expressed by Δ notation [40], in which Δ^H^X_a-b_ = δ^H^X_a_−δ^H^X_b_. Though Δ values are relatively simple to calculate, they become less accurate with increasing differences between the δ values of the substances of interest [41,42]. Because scat isotope values have the potential to be quite different from other tissues, following Passey et al. [43] and Crowley et al. [41], for tissue-to-tissue differences we report the isotope enrichment values (ε) derived from the fractionation factor (α): α_a-b_ = (δ^H^X_a_ + 1,000)/ (δ^H^X_b_ + 1,000) and ε_a-b_ = (α_a-b_− 1) x 1,000. Furthermore, we use the notation ε*, the apparent enrichment value, to denote that this is a non-equilibrium fractionation factor. It is important to note that the sign of enrichment depends on the tissue or substrate in the numerator when calculating α. Scat isotope values are not likely to be considerably different from diet values, however, so we follow the convention in the more recent literature and report “diet-to-scat” discrimination factors as Δ^H^X = δ^H^X_scat_−δ^H^X_diet_ (e.g., [25,44]).

### Gross fecal analysis

Following matrix extraction, we placed each scat in a bag made from nylon panty hose and washed it in a portable automatic washing machine (Haier Compact HLP21N; purchased expressly for this purpose) [1] without detergent to remove any residual matrix and to better separate the remaining components. Once dry, we placed the scat contents on a gridded sorting tray to estimate the percent by volume contributions of mammal, bird, reptile, invertebrate, and plant components to the nearest 5% [45]. While the fur in each scat was spread out, we sampled guard hairs from the center of each grid cell until we had examined 40–50 hairs and identified them to the finest taxonomic level possible through comparison with a guard hair reference collection housed at UC Santa Cruz and with published keys [46–48]. We grouped identified hairs into four categories: marine mammals, small terrestrial mammals (≤ 1 kg), medium terrestrial mammals (> 1 kg, < 30 kg), and large terrestrial mammals (≥ 30 kg). To facilitate comparison with other studies we also calculated the frequency of occurrence of prey taxa as percentage of occurrence = (number of occurrences of prey type/total number of occurrence) x100 [49].

### Data analysis

We used Stable Isotope Analysis in R (SIAR, package ‘simmr’) [50], a Bayesian stable isotope mixing model, to estimate the proportional contributions of various scat components to coyote diets. SIAR is capable of accounting for error in estimates of discrimination factors as well as for variations in the elemental concentrations of C and N in the food sources, which could otherwise bias model output [51]. We derived digestible [C] and [N] values for various coyote food sources through the USDA nutrient database as described by Koch and Phillips [52] (S1 Table). We converted stable isotope values measured in identifiable scat components (S1 Table) to values for muscle tissue, which makes up the bulk of assimilated diet, by applying published organism- and tissue-specific discrimination factors (S2 Table). Using these values, we ran the mixing models both for each scat individually as well as for all the scat samples collectively. Scats were adjusted for trophic discrimination by adding 1.5‰ ± 1.6‰ for δ^13^C values and subtracting 2.3‰ ± 1.3‰ for δ^15^N values (details regarding our arrival at these specific diet-to-scat discrimination factors are explained in the Results and Discussion).

The coyote dietary categories outlined above (bird, invertebrate, etc.) were not necessarily isotopically distinct or homogenous. For example, our subset of scat samples contained feathers from one bird feeding primarily on marine resources and from another feeding primarily on terrestrial resources. For the purposes of the mixing model, rather than combining these isotopically distinct birds into one source (“birds”), we kept them as separate source inputs (“marine bird” and “terrestrial bird”). To enable comparison among the mixing model predictions and those derived from the two GFA techniques, we recast the more numerous mixing model source categories into the eight organism-based dietary categories described above by combining the model predicted mean values. We performed all statistical analyses in R [53].

## Results

### Apparent isotope enrichment factors

**Fig 2:**
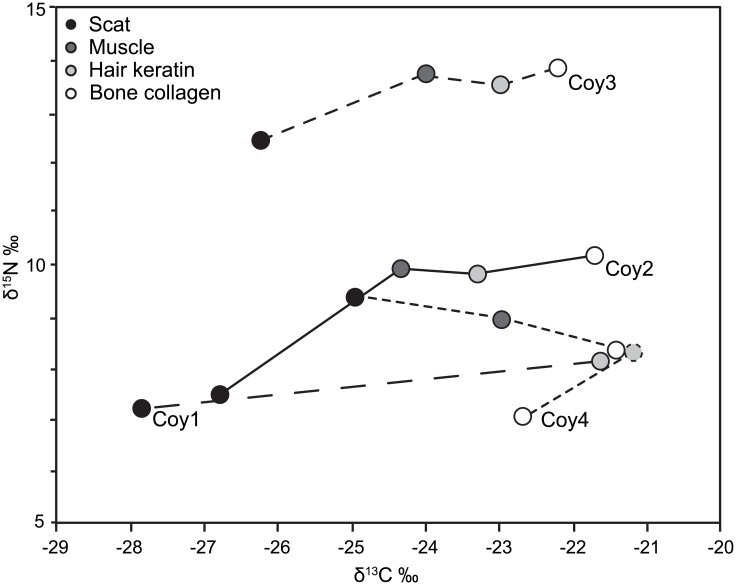
δ^13^C and δ^15^N values measured in tissues of 4 road kill coyotes. Symbol shades denote type of tissue sampled. Tissues from the same individual are connected by lines with different dash patterns.

Carbon isotope values in coyote tissues increased consistently from scat, to muscle, to hair, to bone collagen in the four coyote carcasses we examined (Fig 2, Table 1). Nitrogen isotope values followed roughly the same trend, though the magnitude of change was somewhat less among tissues from a single individual. In addition, muscle δ^15^N values were equal to or slightly higher than hair values. Mean ε^15^* values between proteinaceous tissues were consistently small (≤0.3‰; Table 2), whereas mean ε^13^* values between proteinaceous tissues ranged from -1.0 to 2.3‰. Mean ε^13^* and ε^15^* values between collagen and scat (4.3 ± 2.3‰, 0.9 ± 1.9‰, respectively) as well as between hair and scat (4.1 ± 1.5‰, 0.9 ± 1.3‰, respectively) were higher than ε^13^* and ε^15^* values between proteinaceous tissues (Table 2).

**Table 1:** List of coyote carcasses examined and tissues sample.

Specimen	Sex	Mass (kg)	Age	Year collected	Hair	Bone collagen	Muscle	Feces	Collected by
Coy1	male	11.3	adult	2012	x	x	-	x	R. Reid
Coy2	female	11.5	adult	2012	x	x	x	x	R. Reid
Coy3	male	4.2	juvenile	2012	x	x	x	x	R. Reid
Coy4	male	17.7	adult	2013	x	x	x	x	T. Lambert

**Table 2:** Apparent C and N isotope enrichment factors among sampled tissues of road kill coyote carcasses.

**Specimen**	ε^13^*_collagen-hair_	ε^13^*_collagen-muscle_	ε^13^*_muscle-hair_	ε^13^*_hair-scat_	ε^13^*_collagen-scat_	ε^13^*_muscle-scat_
Coy1	0.2	-	-	6.4	6.6	-
Coy2	1.6	2.7	-1.0	3.6	5.2	2.5
Coy3	0.8	1.8	-1.0	3.3	4.1	2.3
Coy4	-2.0	-0.1	-1.9	3.2	1.2	1.2
**Mean**	**0.2**	**1.5**	**-1.3**	**4.1**	**4.3**	**2.0**
**SD**	**1.5**	**1.4**	**0.5**	**1.5**	**2.3**	**0.7**
	ε^15^*_collagen-hair_	ε^15^*_collagen-muscle_	ε^15^*_muscle-hair_	ε^15^*_hair-scat_	ε^15^*_collagen-scat_	ε^15^*_muscle-scat_
Coy1	0.2	-	-	0.9	1.1	-
Coy2	0.4	0.3	0.1	2.3	2.7	2.4
Coy3	0.3	0.2	0.2	1.1	1.4	1.2
Coy4	-0.9	-1.5	0.6	-0.8	-1.7	-0.2
**Mean**	**0.0**	**-0.3**	**0.3**	**0.9**	**0.9**	**1.1**
**SD**	**0.6**	**1.0**	**0.3**	**1.3**	**1.9**	**1.3**

(ε*): ε_a-b_ = (α_a-b_− 1) x 1,000, and (α): α_a-b_ = (δ^H^X_a_ + 1,000)/ (δ^H^X_b_ + 1,000).

**Fig 3:**
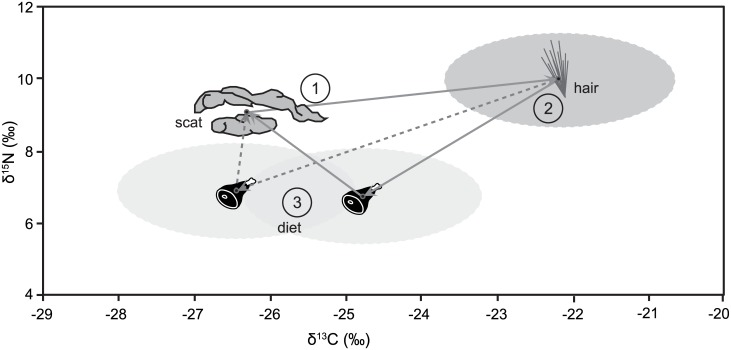
Illustration of derived diet-to-scat C and N isotope discrimination factors for coyotes. The routes through diet-to-hair discrimination factors for red foxes (gray arrows) and through wolves (dashed gray arrows) are depicted. In both cases, the first step used our scat-to-hair ε^13^* and ε^15^* values (4.1 ± 1.5‰, 0.9 ± 1.3‰, respectively) to convert scat to hair; the dark gray oval surrounding the hair point depicts 1 SD around the enrichment factors. In step 2, we used published diet-to-hair enrichment factors for red foxes (C: 2.6 ± 0.4‰, N: 3.2 ± 0.3‰; [26]) and wolves (C: 4.25 ± 0.4‰, N: 3.1 ± 0.2‰; [27]) to convert hair to diet; gray error oval around the diet points depict the propagated standard deviation. Finally, in step 3, we calculated the values necessary to convert from diet to scat.

Given that we chose to work with road kill carcasses, we were not able to directly measure the diets of the individual animals we examined. Therefore, we derived diet-to-scat Δ^13^C and Δ^15^N values for coyotes by relying on our hair-to-scat enrichment factors combined with diet-to-hair discrimination factors determined for red foxes [26] and for wolves [27] (Fig 3). We calculated a diet-to-scat Δ^13^C value of -1.5 ± 1.6‰ and a diet-to-scat Δ^15^N value of 2.3 ± 1.3‰ for coyotes using red fox diet-to-hair discrimination factors from Roth and Hobson [26]. Using muscle-to-scat enrichment factors combined with Roth and Hobson’s [26] diet-to-muscle discrimination factors resulted in the same diet-to-scat discrimination factors for coyotes within error (Δ^13^C = -0.9 ± 0.8‰; Δ^15^N = 2.5 ± 1.3‰). If we instead relied on recently published diet-to-hair fractionation factors for wolves [27], we arrived at a diet-to-scat Δ^13^C value of 0.1 ± 1.6‰ and a diet-to-scat Δ^15^N value of 2.2 ± 1.3‰. We report the diet-to-scat Δ^13^C and Δ^15^N values for coyotes derived through red fox hair (Δ^13^C = -1.5 ± 1.6‰; Δ^15^N = 2.3 ± 1.3‰), as opposed to wolf hair, because these values most consistently place coyote scats within the mixing space provided by known dietary items. Furthermore, we argue that as omnivores, coyote digestive physiology is likely to be more like that of omnivorous red foxes than carnivorous wolves.

### Gross fecal analysis

**Fig 4:**
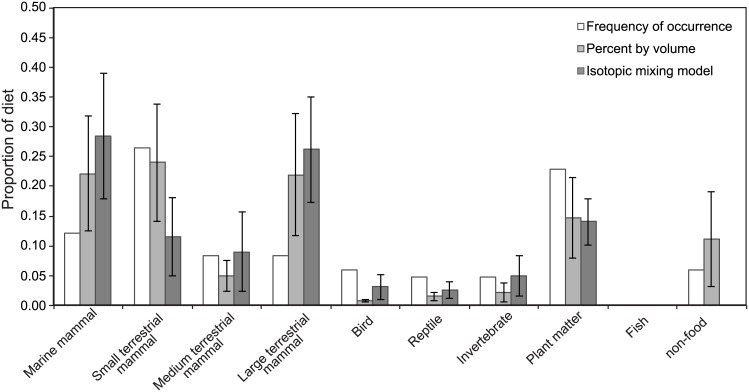
Comparison of diet quantification methods. Comparison of the proportional contributions of marine mammals, terrestrial mammals (small, medium and large), birds, reptiles, invertebrates, plants, fish, and non-food material (e.g., gravel/sand) to 12 DNA-verified coyote scats as identified by three methods: frequency of occurrence (white), percent by volume (light gray) and isotopic mixing models (dark gray). Error bars depict one standard error.

**Fig 5:**
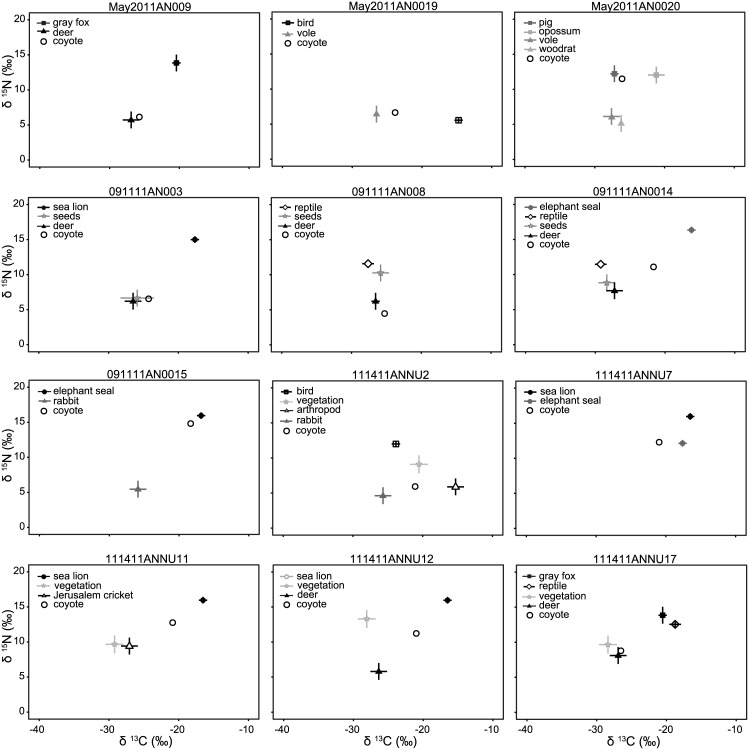
Scat isotope results. Carbon and nitrogen isotope values (δ^13^C and δ^15^N) measured in twelve coyote scats (corrected for discrimination; open circles) from Año Nuevo State Park, CA, plotted in reference to isotope values measured in dietary components found in the scat.

We fully dissected a subset of 12 DNA-verified coyote scats, collected in two different seasons (spring and fall). Because coyote scats are not morphologically distinguishable from other mammalian mesopredator scats in the ecosystem, we previously found DNA-verification to species to be necessary [29]. We identified 25 different dietary components in these scats, including, for example, grass, black-tailed deer (*Odocoileus hemionus*), California sea lion (*Zalophus californianus*), and rabbits (*Sylvilagus bachmani*). The diet estimates derived from the different methods for quantifying coyote diet from scat are similar, but as expected, not identical (Fig 4). Collectively, terrestrial mammals had the highest frequency of occurrence in the scats (43%), with small mammals making up the bulk of that number (27%). These dietary sources were followed by various forms of vegetation (23%) and then marine mammals (12%). Birds, sand/gravel, invertebrates, and reptiles make up the remaining 22%. The percent by volume method similarly identified terrestrial mammals—represented in scat by both fur and bone—as the most frequently occurring coyote scat component (54 ± 13%). Of these, small terrestrial mammals were once again of greatest importance (24 ± 10%). Terrestrial mammals were followed in prevalence by marine mammals (22 ± 8%) and vegetation (13 ± 7%) and the remaining 13% is comprised of sand/gravel, invertebrates, birds, and reptiles. Once adjusted for trophic discrimination, scat matrix stable isotope values fell consistently within the isotope mixing space created by the known dietary components found in them, with two exceptions (samples 091011AN008 and 111411ANNU7) (Fig 5). The aggregated mixing model predictions diverged slightly from the GFA techniques; looking at the mean of the individual model predictions, we saw that terrestrial mammals still contributed the most to coyote diets (47 ± 13%), but small terrestrial mammals accounted for a smaller proportion that approaches significance (12 ± 7% vs. 24 ± 10% in percent by volume; paired t-test: df = 11, *p* = 0.057). Marine mammals also accounted for a higher percentage (28 ± 11%) than predicted by the two GFA methods (12% and 22 ± 8%), though not significantly higher (paired t-test: df = 11, *p* = 0.3).

## Discussion

### Carbon isotope values

Although the carbon in animal tissues is supplied by diet, there are still significant differences in δ^13^C values among tissues [54]. With the exception of Coy4, we found collagen to have the highest δ^13^C values of the tissues we examined, similar to previous studies (e.g., [8,10,55]). Scat, on the other hand, consistently had the lowest δ^13^C values. There was considerable variability in the ε^13^* values between coyote tissues, with values for Coy1 and Coy4 deviating, sometimes in opposite directions, from those for Coy2 and Coy3. Some of this variability may be accounted for by the nutritional state of the individuals we sampled, though none of the carcasses sampled displayed any obvious outward signs of malnutrition or poor health. It has also been previously documented that carbon isotope discrimination among tissues can increase when the δ^13^C values of the dietary carbon sources are variable [56], suggesting that the variability we observe here could in part be driven by the diets these coyotes were consuming. Our sample size of 4 road kill individuals is not likely to be fully representative of the local wild population, however it is comparable to the number of individuals used in many controlled feeding studies [e.g., 17,24,44].

Our estimated diet-to-scat Δ^13^C value for coyotes (-1.5 ± 1.6‰) is of greater magnitude than the experimentally derived diet-to-scat discrimination reported for large mammalian herbivores (-0.8‰; [9]), but consistent in the direction of offset. A previous study found that herbivore scat contained more ^13^C enriched plant acid-detergent fibers than diet items and hypothesized a positive diet-to-feces C isotope discrimination factor [9]. It may be that the microfloral component of scat is the source of the low δ^13^C values, but Sponheimer et al. [9] did not observe an increase in scat δ^13^C following their removal from the sampled feces. Lipids are another known source of light carbon [54], but canids tend to use lipids quite efficiently. For example, Coffey et al. [57] found that fecal excretion of fat by healthy dogs varied from just 2 to 4% and was mostly composed of fatty acids. This could, however, vary significantly with the diet and nutritional state of the animal. Bulk diet-to-lipid discrimination can be quite large (e.g., gerbils = -3‰ [58]; striped skunks = -3.3‰ [59]), and given that scat δ^13^C values are generally lower than diet, but not as low as pure lipids, a small proportion of fatty acids in scat may be enough to account for low scat δ^13^C values. Tiger and snow leopard scat samples that were lipid-extracted prior to analysis were enriched in ^13^C relative to diet [25], strongly suggesting that lipids are responsible for the low δ^13^C values in carnivore scats, though future work on scat δ^13^C values is necessary to definitively resolve the source of the light carbon.

### Nitrogen isotope values

Irrespective of tissue type, δ^15^N values increase with trophic level. This pattern is broadly attributed to the combined effects of (1) higher dietary protein intake with increasing trophic level and (2) the preferential excretion of ^14^N in urea, the main efflux of nitrogen in mammals, resulting in a body pool that is enriched in ^15^N relative to diet [60,61]. Modeling efforts suggest that nitrogen recycling is in part responsible for the ^15^N-enrichment in animal tissues [62]. Nitrogen metabolism is extraordinarily complex [63] and studies continue to demonstrate the intricate connection between isotopic ecology and nutritional ecology (e.g., [64]). A number of controlled feeding studies indicate the magnitude of the ^15^N trophic enrichment correlates with dietary protein content [15,65–67], particularly in animals with high rates of N excretion relative to N assimilation. Other recent work has highlighted the importance of protein quality, suggesting that increasing dietary protein quality corresponds with reduced nitrogen discrimination [68]. In support of this idea, recent studies on fish and birds have shown that as the imbalance in amino acid composition between a consumer and its diet drops (i.e., as diet quality rises), amino acid trophic discrimination factors (which drive bulk diet-to-tissue fractionation) decrease [69,70].

Physiologists recognize two types of "waste" nitrogen lost by vertebrates: endogenous urinary nitrogen (EUN) and metabolic fecal nitrogen (MFN) [64]. EUN is primarily composed of nitrogenous waste products, such as ammonia, uric acid, urea, and creatinine [71]. MFN, on the other hand, is made up of non-absorbed digestive enzymes, intestinal cellular debris, and undigested bacteria and mucus [71]. It is difficult to allocate the contributions of these components to feces as a whole, though previous work suggests that most fecal nitrogen is derived from sloughed endogenous tissues and microbial cells for herbivores [72]. Unlike EUN, fecal δ^15^N values are consistently enriched in ^15^N relative to diet [13,15,17,21], like other tissues. Fecal δ^15^N values elevated above those of diet suggest that the bulk of fecal nitrogen is from the animal itself, rather than undigested food. This hypothesis is supported by an observational study of captive mammalian herbivores, in which the authors found that 60–80% of herbivore fecal nitrogen was endogenous [73]. Omnivores and carnivores, however, do not rely on N-poor plants for protein and their differing digestive physiology may result in differing proportions of endogenous nitrogen in feces. Previously reported diet-to-scat Δ^15^N values are quite variable (S3 Table), ranging from as little as 0.8‰ for cows fed a variety of diets [74] to 3.1‰ for sheep fed alfalfa hay cubes [75]. For six different small mammal species kept on an artificial laboratory diet, diet-to-scat Δ^15^N values varied from 1.4 to 2.6 [44]. These small mammal species (including mice, voles, and a chipmunk; listed in full in S3 Table) have potentially quite different and variable wild diets, however, so it is unlikely that the standard rodent chow used in that study matched the composition of wild diets for all six of the species [56]. Experimentally derived diet-to-scat discrimination factors for two big cats [25] are comparable in direction and magnitude to our results for coyotes. In light of the observed negative relationship between trophic discrimination factors and diet quality [69,70], we hypothesized that diet-to-scat Δ^15^N values would inversely scale with dietary nitrogen intake and therefore would be smallest in carnivores, intermediate in omnivores and greatest in herbivores. The published diet-to-scat Δ^15^N data do not, however, exhibit a significant pattern in terms of trophic level (S2 Fig), either in line with our hypothesis or in contradiction to it. Some of the spread in diet-to-scat Δ^15^N values within trophic groups could be derived from the fact that in many controlled feeding experiments, the animals are allowed insufficient time to come into equilibration with their diets or perhaps because their artificial diets are not of the same quality and/or composition as they are in the wild [56]. Nonetheless, future controlled feeding studies will likely be most effective for elucidating the relationship among dietary protein quantity, quality, and nitrogen discrimination factors.

Given that δ^15^N values increase with trophic level, we anticipated scat matrix δ^15^N values to positively correlate with the amount of protein (meat) predicted to be in the coyote diets by GFA and the isotopic mixing models. While true, the correlations between scat δ^15^N values and the proportion of meat in the diet are weak and not statistically significant (using three different measures of proportion of meat: frequency of animal occurrence [r = 0.18, *p* = 0.57], % volume animal consumed [r = 0.16, *p* = 0.62], and mean proportion animal consumed [r = 0.32, *p* = 0.31; S3 Fig). The same is true for black bear scats [12]. Scat C:N ratios could alternatively be an indicator of an animal’s degree of carnivory, as C:N ratios tend to be high in plants [76,77] and low in animals [78]; because even the fine-grained scat matrix contains food waste in addition to coyote-derived material, animals consuming a largely plant-based diet might produce scats with high C:N ratios and those consuming other animals would then produce scats with lower C:N ratios. We found that scat C:N ratios are significantly negatively correlated with the percent volume of animal material found in the scat (r = -0.71, *p* < 0.01) and also negatively correlated (though not significantly) with the mixing model predicted mean proportion of meat in the coyote diets (r = -0.36, *p* = 0.25; S3 Fig). This lack of a significant relationship lends further support to the idea that the bulk of the scat matrix is composed of material derived from the coyote itself rather than from its food.

### Determining coyote diet from scat

Scats likely capture a short window of food consumption. The average gut retention time for coyotes is on the order of a few days [79], though the incorporation rate of the epithelial cells found in the scat matrix may be longer. Carbon isotope turnover in goat feces following a diet switch was evident in just 2–3 days, however it took 60 days for individuals to reach equilibrium with their diets [80]. At these timescales, stable isotope analyses of scats are particularly useful when working with an organism for which seasonal dietary shifts are important [7,12]. There are other tissues that turn over relatively rapidly (e.g., plasma, breath, ever-growing hair), but their sampling requires physical contact with the animal. Scats provide a non-invasive way to gather short-term dietary information. Furthermore, scats can be linked to individuals either through direct observation (as demonstrated by [7]) or potentially through nuclear DNA analyses [81,82], making it possible to non-invasively monitor individual dietary preferences over time.

The method used to quantify diets from scats is particularly important for omnivores, such as coyotes and foxes; the less uniform an animal’s diet, the larger the disagreement among different methods [1]. Frequency of occurrence tends to over emphasize the importance of small food items in our data—small mammals were overwhelmingly identified as the most important—this result is likely because there are more indigestible parts per unit biomass for small mammals than for larger ones [1,83]. The percent by volume method mitigates small mammal inflation to some degree, but the results are overall quite similar to frequency of occurrence. The stable isotope mixing model results, however, identify larger bodied organisms, such as marine mammals and deer, as more important dietary components. Diet estimates based on biomass calculations also similarly address this bias by placing greater emphasis on larger bodied organisms than frequency of occurrence estimates [1]. While there are fewer indigestible hard parts from large animals in the scat, a substantial proportion of assimilated diet is coming from these animals. It makes sense that mesocarnivores would largely avoid the bones of deer- to pinniped-sized mammals, with the possible exception of fawns and neonate pups, when plenty of more easily consumed and high nutrient soft tissues are available. The stable isotope mixing models also de-emphasize the importance of plant matter to coyote diet. Grass accounted for most of the vegetation identified in these scats. While grass is frequently found in coyote scat, there is no consensus on its role as a food resource; some researchers suggest incidental ingestion while coyotes are capturing prey [84], while others argue it may be a necessary source of vitamins [85]. Our stable isotope mixing model results suggest that grass is less important to assimilated diet, and therefore lends some support to the idea of incidental consumption or some other non-nutritional explanation.

The strength of the stable isotope approach is that scat matrix isotope values provide a faster and less size-biased quantitative estimate of assimilated diet than most methods that rely on quantifying purely undigested material. However, a potential pitfall rests on the degree of isotopic variation in the system; problems will arise if dietary sources are indistinguishable from one another in isotopic space. In the system examined here, a marine resource is one of the major dietary components. Marine systems tend to have much higher carbon and nitrogen isotope values than terrestrial systems. Other sources of variation could come from anthropogenic food sources, which are often C_4_ labeled, or a more diverse flora containing both C_3_ and C_4_ plants. Regardless, questions about resource use may need to be recast, as traditional organism-based dietary categories may not correspond well with isotopic categories. A well-characterized local isotopic baseline will be critical for the interpretation of scat stable isotope values.

We have shown that coyote scat matrix carbon and nitrogen isotope values can serve as proxies for coyote diets. Our derived diet-to-scat discrimination factors are the best approximation available for a wild, mammalian omnivore on a natural diet and better validated than simply applying diet-to-feces discrimination factors observed for herbivores, as done previously [21]. Coyote scat matrix δ^13^C and δ^15^N values consistently plot within the isotopic mixing space created by known dietary sources, suggesting that these discrimination factors are appropriate. Stable isotope mixing model estimates of dietary proportions are complementary, though not identical, to estimates from GFA. Given that the stable isotope mixing model estimates placed greater emphasis on larger-bodied prey items, just as previous authors have noted for biomass calculations, these data suggest that scat stable isotopes provide less size-biased estimates of diet than GFA (given sufficient variation in the ecosystem). Finally, the tissue-to-tissue apparent enrichment factors determined in this study can be applied to wild animals and fossil organisms and will facilitate comparison among isotopic studies performed on a variety of tissue types.

## Supporting information

S1 Fig Test of the effects of rinsing following acidification. δ^15^N values measured in split scat samples that were either rinsed (dark grey) or not rinsed (light grey) after acidification. The mean sample difference is -0.1‰, which is indistinguishable from instrumental error (± 0.1‰).
(EPS)

S2 Fig Δ^15^N by diet type. Boxplots depicting nitrogen discrimination factors for four different types of mammalian feeders: herbivores, omnivores, carnivores and insectivores.
(EPS)

S3 Fig Correlations by dietary proxy. Correlations between scat C:N ratio, δ^15^N and δ^13^C and frequency of animal material occurrence (left column), percent volume animal material (middle column) and mean proportion meat consumed (right column).
(EPS)

S1 Table Mixing model input. Isotope values and digestible [C] and [N] values measured in items identified in each scat sample for input into the mixing models. Food source δ^13^C and δ^15^N values are converted to coyote diet space using discrimination factors listed Table S2.
(DOCX)

S2 Table Coyote diet-space corrections. Organism- and tissue-specific isotope discrimination factors applied to coyote food source δ^13^C and δ^15^N values before input into the SIAR mixing model.
(DOCX)

S3 Table Published diet-to-feces Δ^15^N values in mammals. (DOCX)
