# Supplementary material for: Isotopic ecology of coyotes from scat and road kill carcasses: A complementary approach to feeding experiments
Source: PLoS One. 2017 Apr 3;12(4):e0174897. doi: 10.1371/journal.pone.0174897 (PMC5378380; doi:10.1371/journal.pone.0174897)
Supplement: S1 Table — Isotope values and digestible [C] and [N] values measured in items identified in each scat sample for input into the mixing models. Food source δ13C and δ15N values are converted to coyote diet space using discrimination factors listed Table S2. (DOCX) [file pone.0174897.s004.docx]

| **Scat Sample** | **Food Source** | **Tissue** | **δ^13^C** | ***SD* δ^13^C** | **[C]** | **δ^15^N** | ***SD* δ^15^N** | **[N]** |
| --- | --- | --- | --- | --- | --- | --- | --- | --- |
| May2011AN009 | *Odocoileus hemionus* | hair | -26.91 | 1.0 | 0.54 | 5.72 | 1.0 | 0.14 |
|  | *Urocyon cinereoargenteus* | hair | -20.42 | 0.5 | 0.60 | 13.85 | 1.0 | 0.11 |
| May2011AN0019 | *Microtus californicus* | bone collagen | -26.52 | 0.2 | 0.55 | 6.44 | 1.0 | 0.12 |
|  | *unknown bird* | feather | -14.75 | 0.5 | 0.62 | 5.56 | 0.3 | 0.08 |
| May2011AN0020 | *Microtus californicus* | bone collagen | -27.61 | 1.0 | 0.55 | 6.13 | 1.0 | 0.12 |
|  | *Neotoma fuscipes* | hair | -26.22 | 0.2 | 0.55 | 5.14 | 1.0 | 0.12 |
|  | *Sus scrofa* | hair | -27.18 | 0.5 | 0.65 | 12.22 | 1.0 | 0.07 |
|  | *Didelphis virginiana* | hair | -21.18 | 1.0 | 0.58 | 12.04 | 1.0 | 0.11 |
| 091111AN003 | *Zalophus californianus* | hair | -17.63 | 0.5 | 0.58 | 15.00 | 0.4 | 0.10 |
|  | *Odocoileus hemionus* | hair | -26.50 | 1.0 | 0.54 | 6.21 | 1.0 | 0.14 |
|  | unknown seed | whole crushed seed | -25.91 | 2.0 | 0.48 | 6.66 | 1.0 | 0.02 |
| 091111AN008 | *Odocoileus hemionus* | hair | -26.55 | 0.5 | 0.54 | 6.22 | 1.0 | 0.14 |
|  | *Vaccinium ovatum* | whole crushed seed | -25.83 | 1.0 | 0.45 | 10.27 | 1.0 | 0.01 |
|  | unknown reptile | skin | -27.63 | 0.7 | 0.53 | 11.58 | 0.4 | 0.14 |
| 091111AN0014 | *Odocoileus hemionus* | hair | -27.24 | 1.0 | 0.54 | 7.69 | 1.0 | 0.14 |
|  | *Mirounga angustirostris* | hair | -16.21 | 0.5 | 0.58 | 16.35 | 0.4 | 0.10 |
|  | unknown seed | whole crushed seed | -28.35 | 1.0 | 0.45 | 8.83 | 1.0 | 0.01 |
|  | unknown reptile | skin | -29.22 | 0.7 | 0.53 | 11.47 | 0.4 | 0.14 |
| 091111AN0015 | *Zalophus californianus* | hair | -16.83 | 0.5 | 0.58 | 15.95 | 0.4 | 0.10 |
|  | *Sylvilagus bachmani* | hair | -25.88 | 1.0 | 0.54 | 5.46 | 1.0 | 0.14 |
| 111411ANNU02 | unknown bird | feather | -23.84 | 0.5 | 0.62 | 12 | 0.3 | 0.08 |
|  | *Sylvilagus bachmani* | hair | -25.69 | 1.0 | 0.54 | 4.62 | 1.0 | 0.14 |
|  | unknown vegetation (grass) | crushed leaf | -20.53 | 1.0 | 0.39 | 9.09 | 1.0 | 0.04 |
|  | unknown arthropod | chitin | -15.28 | 1.0 | 0.43 | 5.88 | 1.0 | 0.13 |
| 111411ANNU07 | *Mirounga angustirostris* | hair | -17.61 | 0.5 | 0.58 | 12.14 | 0.4 | 0.10 |
|  | *Zalophus californianus* | hair | -16.51 | 0.5 | 0.58 | 15.96 | 0.4 | 0.10 |
| 111411ANNU11 | unknown vegetation | crushed leaf | -29.07 | 1.0 | 0.39 | 9.65 | 1.0 | 0.04 |
|  | *Stenopelmatus* spp. | chitin | -27.06 | 1.0 | 0.40 | 9.42 | 1.0 | 0.10 |
|  | *Zalophus californianus* | hair | -16.51 | 0.5 | 0.58 | 15.96 | 0.4 | 0.10 |
| 111411ANNU12 | *Odocoileus hemionus* | hair | -26.37 | 1.0 | 0.54 | 5.81 | 1.0 | 0.14 |
|  | *Zalophus californianus* | hair | -16.51 | 0.5 | 0.58 | 15.96 | 0.4 | 0.10 |
|  | unknown vegetation (seaweed) | crushed leaf | -28.09 | 1.0 | 0.38 | 13.3 | 1.0 | 0.04 |
| 111411ANNU17 | *Odocoileus hemionus* | hair | -26.92 | 1.0 | 0.54 | 8.1 | 1.0 | 0.14 |
|  | *Urocyon cinereoargenteus* | hair | -20.54 | 0.5 | 0.60 | 13.85 | 1.0 | 0.11 |
|  | unknown reptile | skin | -18.75 | 0.7 | 0.53 | 12.55 | 0.5 | 0.14 |
|  | unknown vegetation (grass) | crushed leaf | -28.38 | 1.0 | 0.38 | 9.66 | 1.0 | 0.04 |
