# Supplementary material for: Isotopic ecology of coyotes from scat and road kill carcasses: A complementary approach to feeding experiments
Source: PLoS One. 2017 Apr 3;12(4):e0174897. doi: 10.1371/journal.pone.0174897 (PMC5378380; doi:10.1371/journal.pone.0174897)
Supplement: S2 Table — Organism- and tissue-specific isotope discrimination factors applied to coyote food source δ13C and δ15N values before input into the SIAR mixing model. (DOCX) [file pone.0174897.s005.docx]

| **S2 Table.** **Coyote diet-space corrections**. Organism- and tissue-specific isotope discrimination factors applied to coyote food source δ^13^C and δ^15^N values before input into the SIAR mixing model. | | | | | | | | |
| --- | --- | --- | --- | --- | --- | --- | --- | --- |
| **Organism** | $\boldsymbol{\Delta}$**^13^C_keratin -muscle_** | $\boldsymbol{\Delta}$**^13^C_collagen -muscle_** | $\boldsymbol{\Delta}$**^13^C_skin -muscle_** | $\boldsymbol{\Delta}$**^15^N_keratin -muscle_** | $\boldsymbol{\Delta}$**^15^N_collagen-muscle_** | $\boldsymbol{\Delta}$**^15^N_skin-muscle_** | **Citation** |  |
| *Zalophus californianus* | -1.9 ± 0.5 | - | - | -0.6 ± 0.4 | - | - | C: Hobson et al. 1997; N: Hobson et al. 1996 |  |
| *Mirounga angustirostris* | -1.4 ± 0.5 | - |  | -0.6 ± 0.4 |  |  | Hobson et al. 1996 |  |
| Unknown reptile | - | - | -1.3 ± 0.7 | - | - | -1.0 ± 0.4 | C: Warne et al. 2010; N: Seminoff 2009 |  |
| *Urocyon cineroargenteus* | -1.5 ± 0.5 | - | - | no change | - | - | Roth and Hobson 2000 |  |
| Unknown bird | -0.3 ± 0.5 | - | - | -0.6 ± 0.3 | - | - | Hobson and Clark 1992 |  |
| Rodent | -1 ± 0.2 | -2.7 ± 1 | - | -0.8 ± 0.4 | no change | - | keratin: Miller et al. 2008; collagen: Tieszen 1983 |  |
| Rabbit | no change | - | - | no change | - | - | Hilderbrand 1996 |  |
| *Odocoileus hemionus* | -1.6 ± 1 | -4 ± 1 | - | no change | no change | - | keratin: Codron et al. 2007; collagen: Ambrose 1993, Newsome 2004 |  |
| *Sus scrofa* | -1.9 ± 0.5 | - | - | no change | - | - | Nardoto et al. 2006 |  |
