# Supplementary material for: Isotopic ecology of coyotes from scat and road kill carcasses: A complementary approach to feeding experiments
Source: PLoS One. 2017 Apr 3;12(4):e0174897. doi: 10.1371/journal.pone.0174897 (PMC5378380; doi:10.1371/journal.pone.0174897)
Supplement: S3 Table — (DOCX) [file pone.0174897.s006.docx]

| **S3 Table.** Published diet-to-feces **△**^15^N values. | | | | | | | | | | | |  |
| --- | --- | --- | --- | --- | --- | --- | --- | --- | --- | --- | --- | --- |
| **Species** | **δ^15^N_diet_** | **SD** | **δ^15^N_feces_** | **SD** | **△^15^N** | **SD** | **Body Size (kg)** | **Type of Feeder** | **n** | **_citation_** | | |
| *Lama glama (Medicago diet)* | 0.4 | - | 3.3 | 0.3 | 2.9 | 0.3 | 100 - 200 | herbivore | 4 | Sponheimer et al. 2003 | | |
| *Lama glama (Cynodon diet)* | 5.8 | - | 8.8 | 0.4 | 3.0 | 0.4 | 100 - 200 | herbivore | 4 | Sponheimer et al. 2003 | | |
| *Equus caballus* | 0.4 | - | 3.0 | - | 2.6 | - | 200 - 900 | herbivore | - | Sponheimer et al. 2003 | | |
| *Ovis aries* | 0.8 | - | 3.9 | - | 3.1 | - | 10 - 50 | herbivore | 4 | Sutoh et al. 1993 | | |
| *Bos taurus* | 3.0 | 0.1 | 3.8 | 0.3 | 0.8 | 0.3 | 150 - 1300 | herbivore | 10 | Sutoh et al. 1987 | | |
| *Bos taurus* | 0.6 | 0.05 | 2.9 | 1.0 | 2.3 | 1.0 | 150 - 1300 | herbivore | 8 | Steele and Daniel 1978 | | |
| *Microtus longicaudus* | 3.6 | 0.02 | 5.9 | - | 2.3 | - | < 0.1 | herbivore | 5 | Hwang et al. 2007 | | |
| *Tamias amoenus* | 3.6 | 0.02 | 5.0 | - | 1.4 | - | < 0.1 | herbivore | 5 | Hwang et al. 2007 | | |
| *Peromyscus maniculatus* | 3.6 | 0.02 | 5.8 | - | 2.2 | - | < 0.1 | omnivore | 5 | Hwang et al. 2007 | | |
| *Myodes gapperi* | 3.6 | 0.02 | 5.8 | - | 2.2 | - | < 0.1 | omnivore | 5 | Hwang et al. 2007 | | |
| *Microtus pennsylvanicus* | 3.6 | 0.02 | 6.2 | - | 2.6 | - | < 0.1 | omnivore | 5 | Hwang et al. 2007 | | |
| *Zapus princeps* | 3.6 | 0.02 | 5.9 | - | 2.3 | - | < 0.1 | omnivore | 5 | Hwang et al. 2007 | | |
| *Sus scrofa* | 4.6 | 0.3 | 5.8 | 0.2 | 1.2 | 0.4 | 50 - 300 | omnivore | 3 | Sutoh et al. 1987 | | |
| *Myotis myotis (light)* | 5.3 | 0.6 | 7.1 | 1.3 | 1.8 | 1.4 | < 0.1 | insectivore | 3 | Salvarina et al. 2013 | | |
| *Myotis myotis (heavy)* | 12.9 | 1.2 | 15.2 | 2.2 | 2.3 | 2.5 | < 0.1 | insectivore | 3 | Salvarina et al. 2013 | | |
| *Rhinolophus ferrumequinum (light)* | 5.3 | 0.6 | 6.2 | 1.6 | 0.9 | 1.7 | < 0.1 | insectivore | 3 | Salvarina et al. 2013 | | |
| *Rhinolophus ferrumequinum (heavy)* | 12.9 | 1.2 | 13.9 | 0.5 | 1.0 | 1.2 | < 0.1 | insectivore | 3 | Salvarina et al. 2013 | | |
| *Gorilla beringei* | - | - | 3.9 | 0.5 | 0.6 | - | 90 - 200 | omnivore | 29 | Blumenthal et al. 2013 | | |
| *Pan troglodytes* | 4.14 | 0.17 | 5.96 | 0.28 | 1.2 | 2.7 | 30 - 60 | omnivore | 5 | Tsutaya et al. 2016 | | |
| *Canis latrans* | - | - | 9.1 | 2.4 | 2.3 | 1.3 | 5 - 20 | omnivore | 4 | this study | | |
| *Panthera tigris* | 9.0 | 0.73 | 10.5 | 2.0 | 1.6 | 2.2 | 90 - 300 | carnivore | 8 | Montanari and Amato 2015 | | |
| *Uncia uncia* | 9.0 | 0.73 | 11.4 | 1.3 | 2.5 | 1.5 | 25 - 75 | carnivore | 10 | Montanari and Amato 2015 | | |
| Discrimination factors are expressed here as △^15^N values: δ^15^N_scat_ - δ^15^N_diet_. | | | | | | | | | | |  |  |
